# Implementation strategies for occupational therapists to advance goal setting and goal management

**DOI:** 10.3389/frhs.2023.1042029

**Published:** 2023-06-07

**Authors:** Eunyoung Kang, Julie Chen, Erin R. Foster

**Affiliations:** ^1^ Program in Occupational Therapy, Washington University School of Medicine, St. Louis, MO, United States; ^2^ Program in Occupational Therapy, Department of Neurology & Department of Psychiatry, Washington University School of Medicine, St. Louis, MO, United States

**Keywords:** clinician education, chronic conditions and rehabilitation, patient care planning, goals, implementation mapping, implementation science [MeSH], audit & feedback, fidelity

## Abstract

**Background:**

There is a need for an effective evidence-based system to support high-quality goal setting and goal management implementation. We developed a new system for community-based rehabilitation, MyGoals, along with implementation strategies to support occupational therapists (OTs) in its administration. This study evaluates the acceptability, appropriateness, and feasibility of the implementation strategies, *Clinician Education* and *Audit & Feedback*. It also explores whether OTs achieve the change objectives of the MyGoals implementation strategies and MyGoals intervention fidelity.

**Methods:**

This mixed-methods case series study evaluated the MyGoals implementation strategies developed using Implementation Mapping (IM), specifically IM Task 5 – Implementation Outcome Evaluation. Seven OTs and 13 adults with chronic conditions participated in this study. OTs participated in two *Clinician Education* sessions, delivered two MyGoals interventions, and participated in two *Audit & Feedback* sessions. We evaluated the implementation strategies using the Acceptability of Intervention Measure (AIM), Intervention Appropriateness Measure (IAM), Feasibility of Intervention Measure (FIM), and semi-structured interviews and explored the OTs' self-rated MyGoals change objectives achievement and the intervention fidelity using quantitative MyGoals intervention fidelity measures and interviews. Quantitative data were analyzed using descriptive statistics. Qualitative data were analyzed by two independent coders using content analysis.

**Results:**

Seven OTs participated in this study (mean years of professional experience = 9.3, SD = 5.9). *Clinician Education* and *Audit & Feedback* had high AIM (*M *= 17.9, SD = 2.7), IAM (*M *= 17.3, SD = 3.60), and FIM scores (*M *= 17.3, SD = 3). The OTs also had high mean scores on self-perceived achievement of change objectives and intervention fidelity. Qualitative interviews suggested that the time commitment for *Clinician Education* is a key barrier to its acceptability, appropriateness, and feasibility. Participants also provided suggestions on how to improve the strategies (e.g., providing recorded *Clinician Education*, etc.).

**Conclusions:**

The MyGoals implementation strategies are acceptable, appropriate, and feasible to OTs working in community-based rehabilitation. They support OTs in achieving the change objectives necessary to deliver MyGoals completely and competently. Thus, the MyGoals implementation strategies may support clinicians in implementing a theory-based, client-engaged goal setting and goal management for adults with chronic conditions in community-based rehabilitation. This can ultimately help improve the integration of evidence-based interventions into practice.

## Introduction

Theory-based, client-engaged goal setting and goal management is an essential rehabilitation and occupational therapy (OT) practice to provide person-centered care and improve health in adults with chronic conditions (1, 2). It includes the overall collaborative process of educating clients about the goal setting and goal management intervention purpose and the importance of active client engagement during the intervention, guiding them to reflect on their engagement in activities and roles to brainstorm their goals, helping them to formulate personally meaningful goals and relevant plans, and facilitating their reflection on their goal progress and adjustment of their goals and plans (3, 4). Through these processes, occupational therapists (OTs) and clinicians can establish a therapeutic alliance with clients and provide person-centered rehabilitation tailored to each client's goals (3). Clients can also benefit from these processes by becoming intrinsically motivated to achieve their goals and engage more actively in the intervention (5, 6). In turn, this can ultimately help clients achieve better health (5, 6).

However, there are no widely used evidence-based rehabilitation goal setting and goal management for adults with chronic conditions (3). Current goal setting and goal management practice is lacking in comprehensive theory-based interventions and the explicit promotion of active client engagement during the intervention (3). There have been calls for the development of an effective system to support clinicians in implementing theory-based, client-engaged goal setting and goal management to address these research-practice gaps (7).

To meet this need, we used Intervention Mapping to develop MyGoals, a system that guides OTs to administer high-quality goal setting and goal management for adults with chronic conditions in community-based rehabilitation (8–10). MyGoals' ultimate goal is to help clients achieve their personally meaningful rehabilitation goals. To do so, MyGoals provides clinicians with comprehensive theory-based structured activities that guide them to easily deliver key goal setting and goal management-related components in clinical practice. MyGoals also provides clinicians with scripts designed to facilitate the use of empowerment-based approaches to promote active client engagement during the intervention such as explicitly asking the client's perspectives, desires, and needs to guide decision-making. Supplementary Material S1 provides a sample of the MyGoals manual and client worksheet examples.

Effective implementation strategies are key to supporting clinicians in implementing complex interventions like MyGoals in practice (11). In the absence of effective implementation strategies, clinicians may have difficulty delivering interventions as intended (11). Implementation theories, models, and frameworks can guide implementation strategy development and evaluation (12).

Therefore to support future MyGoals' implementers (OTs), we developed MyGoals implementation strategies using Implementation Mapping (11), the Consolidated Framework for Implementation Research (13), a taxonomy of behavior change (14), social cognitive theory (15), and Proctor's implementation research framework (16). For details on how we developed the strategies using these theories, models, and frameworks and MyGoals implementation determinants, please refer to Kang and Foster (17) and Kang et al. (18). We developed two MyGoals implementation strategies - *Clinician Education* and *Audit & Feedback* – because these fit well with the current pre-implementation stage of MyGoals. Briefly, *Clinician Education* teaches OTs the theoretical background of MyGoals and how to implement it in practice, and *Audit & Feedback* provides OTs with information to help them become aware of their MyGoals intervention delivery quality and improve their knowledge, skills, and self-efficacy to enhance MyGoals intervention implementation.

This study aimed to evaluate the acceptability, appropriateness, and feasibility of MyGoals *Clinician Education* and *Audit & Feedback* for OTs using quantitative measures and qualitative interviews. We hypothesized that the MyGoals implementation strategies would have good acceptability, appropriateness, and feasibility. We also examined OTs' self-perceived achievement of the change objectives of the MyGoals implementation strategies and their MyGoals intervention adherence and competence (intervention fidelity).

## Materials and methods

### Study design

This was a mixed-methods multiple case series study using Implementation Mapping (IM) task 5 – evaluation of implementation outcomes (11). IM is a systematic approach to guide the development and evaluation of implementation strategies. This approach includes the following five tasks: (1) assessment of needs and assets, (2) creation of a logic model of change, (3) design implementation strategies, (4) production of implementation strategies, and (5) evaluation of implementation outcomes (11). In a prior study, we accomplished IM tasks 1 through 4 (17). In the present study, we have completed the 5th IM task, which involves the evaluation of implementation outcomes. We reported our findings using the Standards for Reporting Implementation Studies statement (Supplementary Material S2) (19).

**Figure 1:**
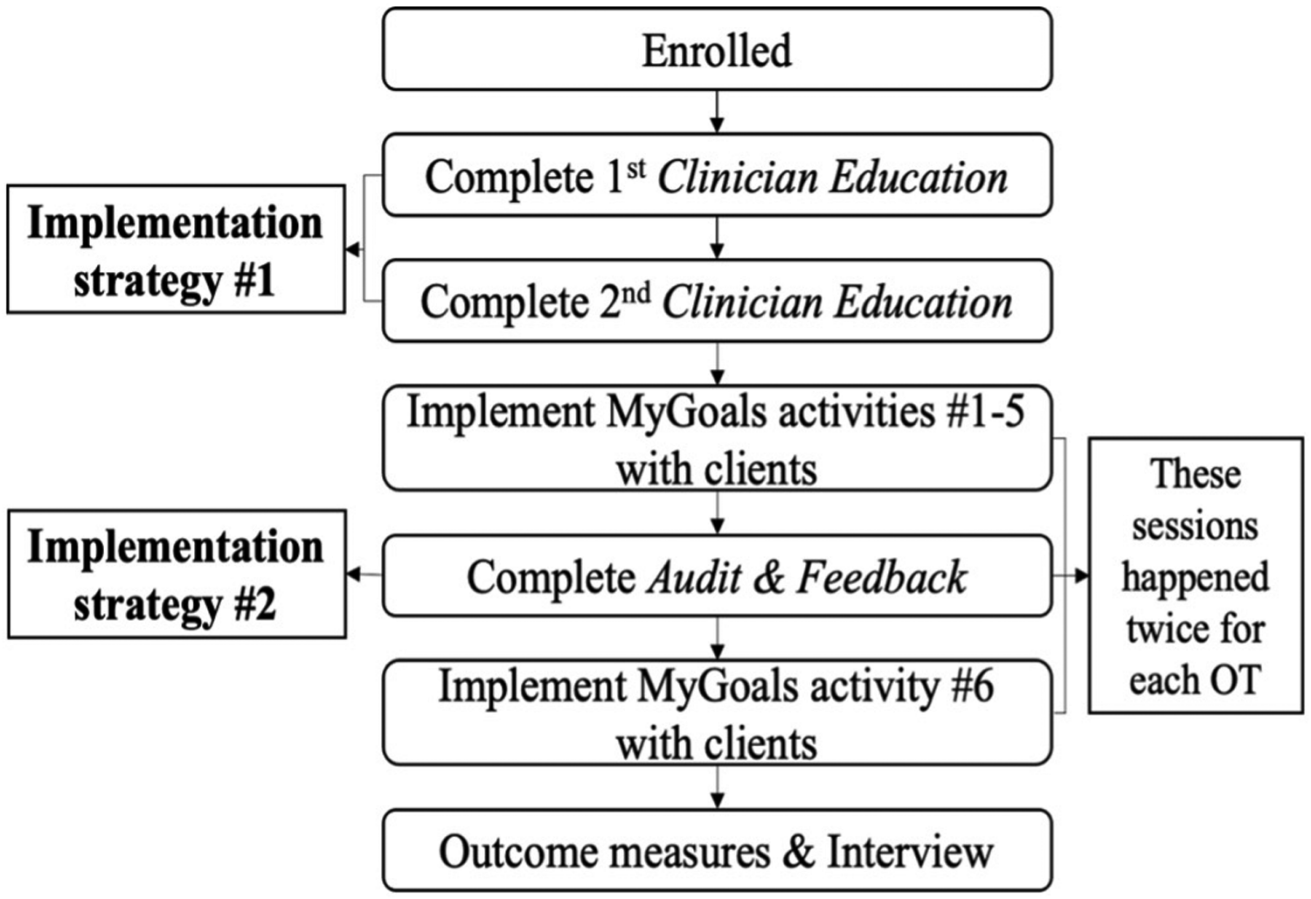
Study flowchart.

Figure 1 describes the overall study flow. OTs completed the following tasks: (1) two 2-h Zoom MyGoals *Clinician Education* sessions with the OT research team member, (2) in-person delivery of MyGoals intervention activities 1. *Education*, 2. *Reflection*, 3. *Find My Goal*, 4. *Make My Goal*, and 5. *Make My Plan*. to the first client, (3) in-person or virtual *Audit & Feedback* session with the OT research team member, (4) in-person delivery of MyGoals intervention activity 6. *My Progress* to the first client, (5) in-person delivery of MyGoals intervention activities 1–5 to the second client, (6) in-person or virtual *Audit & Feedback* session, (7) in-person delivery of MyGoals intervention activity 6 to the second client, and (8) complete outcome measures with the research team.

We included clients who (1) were over the age of 18, (2) had one or more chronic conditions, and (3) did not have severe cognitive or communication impairment operationally defined as having a total Montreal Cognitive Assessment (MoCA) score of 20 or lower (20) through word of mouth, snowball sampling, existing research registries, flyers, and referrals. Clients were involved in two in-person MyGoals intervention sessions. In the first session, they completed MyGoals intervention activities (1) *Education*, (2) *Reflection*, (3) *Find My Goal*, (4) *Make My Goal*, and (5) *Make My Plan*. In the second session, they completed MyGoals intervention activity (6) *My Progress*. The research team randomly assigned OT-Client dyads based on their availability.

Thirteen clients were involved in this study. Nine were female, and four were male. Two participants self-identified as multi-racial (one as American Indian or Alaska Native and White and the other as Black and White). The other 11 participants self-identified as White. Twelve people self-identified as Not Hispanic or Latino, and one did not know their ethnicity. All 13 participants had more than a high school education. The mean total MoCA score was 23.9 (SD = 2.6, range = 21–30). Clients had a wide variety of chronic conditions, and they often had more than one condition (arthritis = 2, diabetes = 1, heart failure = 1, hypertension = 2, multiple sclerosis = 1, Parkinson's disease = 3, schizophrenia = 1, osteoporosis = 1, nerve damage = 1, Crohn's disease = 1, rheumatoid arthritis = 2, basal cell cancer = 1, osteopenia = 1, heart disease = 1, and gout = 1).

### Context

This study was conducted using Zoom and in-person meetings at a research-based university in the Midwest, USA. This study is approved by the university's institutional review board (IRB #: 202107053). All participants provided consent before their participation.

### Participant eligibility and recruitment

We recruited OTs who (1) were over the age of 18, (2) were licensed OTs, and (3) had at least 1-year clinical experience in goal setting and goal management with adults with chronic conditions in community-based rehabilitation settings using word of mouth, snowball sampling, flyers, and Facebook posting.

### MyGoals (clinical intervention)

In our previous studies, we developed MyGoals using Intervention Mapping and its implementation strategies using Implementation Mapping tasks 1–4 in collaboration with client and OT partners (17). In this study, the MyGoals intervention included two weekly in-person sessions. The MyGoals intervention's ultimate goal is to enable clients to achieve personally meaningful rehabilitation goals. It includes six structured activities: *Education, Reflection, Find My Goals, Make My Goals, Make My Plans*, and *My Progress*. Throughout the intervention, the OT guides the client to understand the overall intervention purpose and their expected roles during the intervention, reflect on their current engagement in personally meaningful activities and roles, develop goals and plans, and review and adjust them. To facilitate these processes, MyGoals encourages OTs to use an empowerment-based approach. This approach involves using open-ended questions, using plain language, not demanding clients to change their responses, and explicitly asking about clients' needs, preferences, perspectives, or desires.

### MyGoals implementation strategies

We developed MyGoals implementation strategies using Implementation Mapping. Table 1 describes the *Clinician Education* and *Audit & Feedback* implementation strategies based on the recommendation by Proctor et al. (23).

**Table 1:** MyGoals implementation strategy specification.

	Clinician Education	Audit & Feedback
Actors	The OT research team	The OT research team member who observed and evaluated the OT participant's MyGoals intervention sessions in real-time
Actions	Before MyGoals clinician education 1. Emailed the developed easy-to-understand, eye-catching education materials to the OT to provide the opportunity to review MyGoals intervention activities and their strengths & evidence and relative advantages First 2-h 1 : 1 zoom MyGoals clinician education 1. Introduced MyGoals intervention concepts, importance, activities, strengths & evidence, relative advantages, flexibility 2. Educated on knowledge and skills necessary to deliver MyGoals completely and competently 3. Provided audio-recording of an experienced OT delivering MyGoals to learn ideal MyGoals practice, gain awareness about one's practice, and perceive the potential benefits of using MyGoals 4. Discussed the audio-recording of the experienced OT's MyGoals sessions During the 2nd 2-h zoom MyGoals clinician education 1. Role-played to practice how to translate their knowledge and skills into practice, improve one's self-efficacy to deliver MyGoals, and provide a chance to perceive the relative advantages and strengths & evidence of MyGoals	Audit 1. The research team audited OT's in-person MyGoals intervention sessions with clients in real-time 2. During the observation, the research team evaluated the adherence and competence of OT-delivered MyGoals intervention sessions using the observer-rated MyGoals fidelity assessment - Competence and Adherence Scales Feedback 1. The research team encouraged the OT to share self-reflection on their MyGoals intervention delivery and encouraged them to ask questions 2. The research team discussed the OT's self-reflection and answered questions 3. The research team verbally debriefed the MyGoals fidelity assessment results. The research team made sure to provide positive reinforcement by emphasizing the well-implemented components and communication strategies 4. The research team asked the OT to share their reflections and questions regarding the Fidelity assessment results 5. The research team nonjudgmentally and interactively discussed with the OT ways to improve their knowledge, skills, and performance. The research team helped the OT improve their knowledge, skills, and performance 6. The research team suggested clear target intervention components and communication strategies that can or need to be improved to adhere to the MyGoals manual 7. The research team verbally provided tailored action plans for each OT to help them adhere to the MyGoals manual and improve intervention quality 8. The research team reminded the OT that they now have the necessary knowledge and skills and supported them to enhance their self-efficacy to deliver MyGoals completely and competently
Action target	• OT's knowledge, awareness, skills, outcome expectancy, self-efficacy • MyGoals intervention’ strengths & evidence, and relative advantages	• OT's knowledge, awareness, skill, and self-efficacy
Temporality	Two 2-h zoom sessions provided before the OT delivers MyGoals to clients	Provided between the 1st and 2nd MyGoals intervention sessions with each client (2 sessions per OT participant)
Dose	Two 2-h zoom sessions	Two 0.5-h sessions
Implementation outcomes affected	Acceptability, appropriateness, feasibility, MyGoals intervention fidelity	Acceptability, appropriateness, feasibility, MyGoals intervention fidelity
Justification	We developed the tailored strategies to target the MyGoals implementation determinants, which we identified through a rigorous process (21) including Implementation Mapping (11), a taxonomy of behavior change (14), CFIR (13), and social cognitive theory (15) and based on the existing literature such as ERIC-recommended strategies (22).	

### Outcomes

#### Acceptability, appropriateness, feasibility of MyGoals implementation strategies

We adopted Proctor et al. (16) to conceptually define implementation outcomes. Acceptability was defined as OTs' perception of the extent to which *MyGoals Clinician Education* and *Audit & Feedback* are agreeable, palatable, or satisfactory (16). Appropriateness was defined as the OTs’ perception of the fit, relevance, or compatibility of the strategies to equip them to deliver MyGoals as intended (16). Feasibility was defined as the OTs' perception of the extent to which the strategies can be successfully conducted in community-based rehabilitation (16). We measured these outcomes using the Acceptability of Intervention Measure (AIM), Intervention Appropriateness Measure (IAM), and Feasibility of Intervention Measure (FIM), respectively (24). We used scale scores of ≥16 as predetermined benchmarks for good outcomes. This is because item scores of 4 and 5 suggest that the respondent agrees or completely agrees that MyGoals implementation strategies are acceptable, appropriate, or feasible.

We also explored OTs' perspectives on the acceptability, appropriateness, and feasibility of *Clinician Education* and *Audit & Feedback* using 1-h individual semi-structured interviews. We developed a semi-structured interview guide to explore the barriers of *Clinician Education* and *Audit & Feedback* (Supplementary Material S3). The facilitators of *Clinician Education* and *Audit & Feedback* were not explored, as this study aimed to identify ways to overcome the barriers to improve the implementation strategies. Example questions include “*What aspects of MyGoals Clinician Education and Audit & Feedback were less feasible*?” and “*How can we make MyGoals clinician training more feasible?*” The interviews were audio-recorded and transcribed verbatim.

### Self-perceived achievement of change objectives

We evaluated the 15 change objectives developed in the previous Implementation Mapping Task (17) using an 11-point scale (0: strongly disagree – 10: strongly agree); e.g., “*I understand goal setting and goal management practice concepts and their importance*.” Table 2 lists all questions. If participants rated any item lower than 7, we asked the following interview question to explore how we can improve the implementation strategies to address that change objective [e.g., “*How can we improve MyGoals training so that it can help you to (understand goal setting and goal management practice concepts and their importance)]*?

**Table 2:** Self-perceived change objectives achievement results.

	Change objective item	Mean, SD, range
1	I understand goal setting and goal management practice concepts and their importance	9.7, 0.5, 9–10
2	I understand the MyGoals evidence	8.7, 2, 5–10
3	I acknowledge that current goal setting and goal management practice is not optimal	8.7, 2, 5–10
4	I acknowledge that MyGoals is acceptable	9.3, 1, 8–10
5	I acknowledge that MyGoals is appropriate	9.0, 1.5, 7–10
6	I acknowledge that MyGoals is feasible	8.3, 2, 6–10
7	I expect delivering MyGoals will improve personally meaningful goal achievement in clients	8.0, 2.3, 5–10
8	I understand all MyGoals intervention components	8.5, 1.8, 6–10
9	I have skills for delivering all MyGoals intervention components completely	8.7, 2, 5–10
10	I expect delivering all MyGoals intervention components will improve personally meaningful goal achievement in clients	8.7, 1.8, 6–10
11	I am confident in my ability to deliver all MyGoals intervention components	8.5, 2.1, 5–10
12	I understand 4 MyGoals communication strategies*	8.8, 1.8, 6–10
13	I have skills for delivering all MyGoals activities by using 4 communication strategies*	9.0, 1.4, 7–10
14	I expect using 4 MyGoals communication strategies will improve personally meaningful goal achievement in clients*	9.2, 1.1, 8–10
15	I am confident in my ability to deliver all MyGoals activities by using 4 communication strategies*	8.6, 1.7, 6–10

* *n* = 5.

### MyGoals intervention fidelity

We assessed two aspects of intervention fidelity: adherence and competence (25). We defined adherence as the extent to which the OT implements the MyGoals intervention components (i.e., completeness of delivery) and competence as how well the OT implements the MyGoals empowerment-based approaches during the intervention (i.e., quality of delivery) as observed by the OT research team member (25). The researcher, who is also an OT, rated adherence and competence in real-time using the observer-rated MyGoals Fidelity tool. Adherence was rated using a dichotomous response (yes/no). Competence was rated using a 3-point scale (1 = low, 2 = medium, and 3 = high quality). Table 3 list the MyGoals Fidelity tool items.

**Table 3:** MyGoals intervention fidelity competence results.

MyGoals intervention fidelity competence items	*M*	SD
Used open-ended questioning	2.8	0.5
Used plain language	3.0	0
Explicitly asked the client about their needs, preferences, perspectives, or desires	2.8	0.5
Did not demand the client to change their responses to questions, goals, or plans	2.9	0.2

### Analysis

We calculated frequency, mean, and SD to analyze quantitative outcomes and demographics. To analyze qualitative interview data, we used conventional content analysis (26, 27). Two coders (E.K and J.C) first independently read the transcripts word for word to understand the overall transcript data, highlighted keywords, identified initial codes, and determined codes and categories using the Microsoft Excel program. Then the two coders discussed the analysis results and defined categories and codes to reach a consensus. When there were discrepancies, they were solved through an interactive discussion or consultation with a senior author (E.F.). Quantitative and qualitative data were simultaneously collected and analyzed (QUAN + QUAL) for the complementary purpose (28). We used quantitative data to assess the appropriateness, acceptability, feasibility of MyGoals implementation strategies. We used qualitative data to obtain a comprehensive understanding of the OTs' perspectives on enhancing the MyGoals implementation strategies (28). We addressed trustworthiness, specifically credibility, dependability, and transferability, throughout this study (26). We enhanced the credibility of the data by having a consistent data analysis process in two coders and identified codes and categories based on rich and meaningful data. We improved the dependability of the data by having carefully crafted structured interview questions and two independent coders and a senior author. We enhanced the transferability of the data by describing the detailed analysis results to improve the applicability of our findings across contexts.

## Results

### Participant characteristics

Seven OTs participated in this study. Participants self-reported their sex, race, ethnicity, and years of professional experience. All OTs self-identified as female, White, and not Hispanic or Latino. The mean years of professional experience in rehabilitation were 9.3 (SD = 5.9).

### Acceptability, appropriateness, feasibility of MyGoals implementation strategies

Table 4 shows the AIM, IAM, and FIM results. The mean scores of all scales ranged from 17.3 to 17.9, which is higher than our predetermined benchmark for good acceptability, appropriateness, and feasibility. The item-level mean scores ranged from 4 to 4.6, indicating that respondents perceived all individual-level items of acceptability, appropriateness, and feasibility were high quality.

**Table 4:** Quantitative results on the acceptability, appropriateness, and feasibility of the MyGoals implementation strategies.

AIM, IAM, FIM items	Mean, SD, range
MyGoals training meets my approval	4.6, 0.5, 4–5
MyGoals training is appealing to me	4.4, 0.8, 3–5
I like MyGoals training	4.2, 1, 3–5
I welcome MyGoals training	4.6, 0.5, 4–5
Total AIM score	17.9, 2.7, 14–20
MyGoals training seems fitting	4.3, 1, 3–5
MyGoals training seems suitable	4.3, 1, 3–5
MyGoals training seems applicable	4.3, 1, 3–5
MyGoals training seems like a good match	4.4, 0.8, 3–5
Total IAM score	17.3, 3.6, 12–20
MyGoals training seems implementable	4, 1.3, 2–5
MyGoals training seems possible	4.6, 0.5, 4–5
MyGoals training seems doable	4.6, 0.5, 4–5
MyGoals training seems easy to follow	4.1, 0.9, 3–5
Total FIM score	17.3, 3, 14–20

Participants did not mention any less acceptable, appropriate, and feasible aspects of *Audit & Feedback* or ways to improve that implementation strategy. Participants mentioned that the only less acceptable and feasible aspect of the implementation strategies was the time commitment for two 2-h *Clinician Education* sessions. However, participants also explained that it was worthwhile to invest time to learn new knowledge and skills.

“It was a little bit time consuming to do like the training just with like other stuff (with my regular full-time job) going on … I also feel like … I could have used a little more training … .Maybe it's a little less feasible because it is more time intensive to train, but I also think that's kind of like what you need, so a little like give and take” – OT5.

Participants suggested several methods to improve the acceptability of *Clinician Education* and MyGoals manual and how to mitigate these real-life barriers. To improve *Clinician Education*, participants provided suggestions on three aspects: structure, delivery, and content. To improve *Clinician Education* structure, participants recommended streamlining audio examples and providing audio examples not all at once, but rather throughout the training.

“If it was like shorter clips with like a more specific … targeted communication because I think that part was maybe the only thing that was a little bit hard to focus on” – OT6.

“Spacing out the audio clips to give examples throughout, like as we're initially going through the manual to maybe hear some examples relevant to certain topics” – OT6.

To improve *Clinician Education* delivery, providing a flexible delivery model was recommended. The specific recommendations included providing educational materials before the first session, using a different and/or flexible training mode, providing recorded sessions, and providing quizzes so that OTs can learn at their own pace and time.

“Being able to read through it before people first might be helpful” – OT2.

“The different modes of training are good too. You had some visuals and you had the audio clip and all that just helps make it a little more engaging … I think giving the independent work is good. Because I think people could take it and do it if they have a cancelation. They could do some of the reading or something … An a la carte type of service … do the training as they're able” – OT6.

“I think the recorded sessions would really fit well into that. Because it (3rd party education system) has … courses where you go through PowerPoint slides and things like that. They show like a demonstration of how to implement it. That's super helpful to have both of those components” – OT3.

To improve *Clinician Education* content, participants suggested clarifying communication strategies, briefly addressing common OT skills, taking a deeper look at more advanced skills, providing more intense education on practical skills and knowledge, and offering practical role-playing examples. Participants also emphasized the need to enhance the adaptability of the MyGoals packet.

“I know it talked about the four communication styles or techniques, and I was trying to think … I don't think I could rattle off like it. I don't feel like it was like really made obvious. We're looking at these four styles of communication. I could think about well we did this and this, but it might help to just like, have a section and say this is how we're communicating, and list out those four techniques or styles” – OT6.

“Somethings were pretty inherent to OTs … like asking open-ended questions … Briefly touching on things like that, but then kind of like saving time there and going on to. The most different part of the intervention for me was just kind of learning to take a step back … I try to do this in my practice, too. I feel like it was very emphasized here and for good reason to let the patient kind of take the reins on creating the goal completely, which is great. I don't always do it to that extent because of time purposes. But for somebody who's able to do that, like cognitively, I feel like that's really helpful. So having, like emphasis on that, instead of maybe some of the other stuff that most people know about already might help” – OT3.

“I feel like we probably could have gotten away with doing a little less about the background of it … more of the actual … how do you like put this into practice and like challenges that you might encounter when doing it?  I think that was actually like a really good like … this is how I go to practice because like when we were doing like the background of it and all that stuff, I was like, Yes, this makes perfect sense. And then I had to do the role play and I was like, What now? I don't know where to go from here” – OT5.

“More role-playing with challenging answers” – OT6.

“Just the organization of the manual” – OT3.

To improve the appropriateness of *Clinician Education*, participants suggested improving its structure by providing group sessions, its content by providing tailored training for each clinician and more role-playing, its delivery by providing diverse educational material types, and streamlining the MyGoals manual.

“I think (group education) could be helpful because hearing other people ask those questions and that the gray questions know that aren't like part of the script, but you need to ask to hear somebody else ask” – OT4.

“Make sure that people have … a baseline level of understanding of some of these occupational therapy skills and how to apply them. Since people are bringing different levels of experience into this process” – OT6.

“More role-playing” – OT6.

“Having like a link to like an audio PowerPoint with this like that you have already … everyone learns differently. Having that is a way to just to go through it” – OT4.

“Have like a really concise … handout of … a short blurb about what this is. This is how you do it … kind of jogging your memory … a quick guide to me to know how to do … the intervention … It's easily implemented just to kind of help educate therapists. On the go, basically … Make MyGoals intervention packet easier to follow” – OT3.

To improve the feasibility of *Clinician Education*, participants suggested improving its structure by providing quizzes during the recorded sessions and clinician incentives, and its delivery by providing recorded sessions to allow OTs to complete the sessions at their convenience.

“Having quizzes and things like that to kind of check understanding because a lot of times those components really help … Quiz questions … stick in your mind because you're like should have paid attention to that” – OT3.

“I hate to sound like that let it serve a purpose for me, but like that is a nice thing that you… (provided compensation). I (hope to) also get some CEU (continuing education unit) for it” – OT5.

“Having it be recorded. So that we don't have to coordinate schedules (for the education with a researcher” – OT3.

### Self-perceived achievement of change objectives

Six participants rated their self-perceived achievement of the MyGoals implementation change objectives (Table 2). One participant declined to answer items #12–15 and did not disclose the reason. The mean scores of all items ranged from 8 to 9.7, indicating participants successfully achieved all predetermined change objectives. There were several items with lower minimum responses such as 5 and 6. Table 5 displays these items and the OTs' recommendations on how to improve the implementation strategies to help them achieve these change objectives.

**Table 5:** Qualitative feedback to improve the implementation strategies to address the change objectives with lower self-perceived achievement scores.

Change objective	How can we improve the MyGoals implementation strategies to help you achieve the change objective?
I understand the MyGoals evidence	• Provide empirical evidence (“*I understand the evidence of MyGoals… obviously the principles behind it. You shared that and I understand that, but I feel like the evidence of MyGoals itself is still like in the works*”)
I acknowledge that MyGoals is feasible	• Provide an electronic version MyGoals & streamline MyGoals (“*I feel like if it was like electronic or accessible by computer through like smart phrase and also just it needs somehow has to be shortened or broken up into sessions”)*
I expect delivering MyGoals will improve personally meaningful goal achievement in clients	• Promote tailored MyGoals intervention for each client (“*I feel like for most people, it [MyGoasl intervention] would be really meaningful, but like one system does not work for every person… You have to look at your clients and see who will be really benefit from it and who needs a different approach…Anybody that is goal-oriented would really benefit from this depending on how they do, people that are less goal-oriented could do well from this or could not do well from this because some people either get stressed out by their goals*”)
I expect delivering all MyGoals intervention components will improve personally meaningful goal achievement in clients	• Promote tailored MyGoals intervention for each client (“*There are MyGoals intervention components that are super helpful with goal achievement, but I feel like using all of them all the time, like I was focused on the word “all” there, it is not like feasible all the time. Using the principles is great. I just don't think it's feasible to do this whole process with all the clients. But if it says, like just said “utilizing components from my goals in practice will be personally meaningful in goal achievement with clients” I would say 100 percent that would be like there are so many good components*”)
I am confident in my ability to deliver all MyGoals intervention components	• Improve MyGoals manual adaptability (“*I don't think any of it was like very complicated, necessarily. I just think like I may have gotten a little confused trying to follow the script and use the handouts. Maybe… the way that that packet is organized. It might be helpful to kind of like reorganize it a little bit…So I think that was probably why I put a little bit lower on my confidence*”)
I am confident in my ability to deliver all MyGoals activities by using 4 communication strategies*	• Clarify the communication strategies and provide a cheat sheet (“*I wasn't really quite sure the other four communication strategies, exactly what those were…Provide a cheat sheet for clinicians to implement communication skills*”) Provide more role-playing (“*Having more role playing or being able to like, you know, look at that stuff a little*”)
I understand all MyGoals intervention components	• Provide more practice and role-playing sessions (“*That's just like my skills, I'm not great at… open-ended. That's more like me making my brain do something that it's not like that. I don't know if that's necessarily a problem with the tool…I feel like it was the actual role-playing. Just being able to like maybe do more of that because I even felt like during my the second client, it was like this is at least a little easier than the first time doing it.*”)
I have skills for delivering all MyGoals intervention components completely	• Provide more practice and role-playing sessions (“*That's just me being bad at open-ended things*”)

### MyGoals intervention fidelity

All seven of the OTs were assessed for fidelity with their clients. One OT participant only saw one client, so a total of 13 fidelity assessments were completed. One client was lost after their first visit, so the items in activity 6 for this client were not completed.

For adherence, about 70% of the MyGoals intervention components were implemented by all therapists every time (Table 6). Two components in activity 4 (*Educate and discuss one's current health condition from the biopsychosocial perspective* and *Educate and discuss the benefits of using life goals, goals, and building block goals*) were most commonly omitted.

**Table 6:** MyGoals intervention fidelity adherence results.

[MyGoals intervention activity #] Fidelity - Adherence items	Implemented (%)	Not implemented (%)
[1] Educate and discuss the overall goal setting and goal management concept	100	0
[1] Educate and discuss the client's expected roles during goal setting	100	0
[2] Guide reflection about the current engagement in meaningful activities and roles	100	0
[3] Rate the importance of potential goal activities	100	0
[4] Guide life goal formulation	92.3	7.7
[4] Guide goal formulation	100	0
[4] Guide the self-efficacy level evaluation	100	0
[4] Guide the positive outcome expectancy level evaluation	92.3	7.7
[4] Guide building block goal formulation	100	0
[4] Educate and discuss one's current health condition from the biopsychosocial perspective	69.2	30.8
[4] Educate and discuss the benefits of using life goals, goals, and building block goals	76.9	23.1
[5] Guide barrier identification	100	0
[5] Guide facilitator identification	92.3	7.7
[5] Guide planned behavior identification	100	0
[5] Guide if (when)-then plan formulation	100	0
[5] Guide the self-efficacy level evaluation	92.3	7.7
[6] Educate and discuss client's expected roles during goal management	100	0
[6] Guide goal progress (performance) evaluation	100	0
[6] Guide satisfaction with a goal progress evaluation	100	0
[6] Discuss goal progress	100	0
[6] Discuss Goal And Plan Adjustment	100	0

The MyGoals intervention fidelity competence results are in Table 3. All of the therapists *used plain language* with high quality consistently, with a mean score of 3. Overall, the therapists successfully used the other skills as well, as demonstrated by the near-perfect average scores ranged from 2.8 to 2.9. One participant demonstrated low quality with using open-ended questioning and explicitly asking about the client's needs, preferences, perspectives, or desires.

In addition, the OTs' personal determinants such as existing knowledge and skill, preferences, and past working experiences may have affected the current study findings. This is illustrated by the following interview quotes: “That's just like my skills, I'm not great at open-ended. That's more like me making my brain do something that it's not like that. I don't know if that's necessarily like a problem of the tool”, “Since people are bringing different levels of experience into this process,” “Just my personal preference would be to have like one piece of paper”, etc. The OTs also mentioned that client-related factors (e.g., goal-oriented individuals vs. not goal-oriented individuals) could affect the implementation outcomes.

## Discussion

This study evaluated the acceptability, appropriateness, and feasibility of the MyGoals implementation strategies that we developed and specified using Implementation Mapping (17). We found that MyGoals implementation strategies had high acceptability, appropriateness, and feasibility among a sample of OTs working in community-based rehabilitation. In addition, the OTs perceived that they successfully achieved the change objectives of the strategies and demonstrated good MyGoals intervention fidelity. Our results support the potential of the MyGoals implementation strategies to enhance goal setting and goal management practice in rehabilitation.

The MyGoals implementation strategies were considered acceptable, appropriate, and feasible by OTs. These three outcomes are leading indicators of successful implementation (24). Assessing them in feasibility studies is particularly informative as it provides the stakeholders' perspectives on, and can guide potential modifications of, the strategies to understand and optimize their uptake by the end-users (24). The high acceptability, appropriateness, and feasibility scores in our study demonstrate that the current strategies fit well with the therapists' needs and preferences and suggest they are likely to be used in clinical practice.

The self-perceived change objectives achievement and fidelity data support the potential of the MyGoals implementation strategies in promoting effective goal setting and goal management. The high self-perceived change objective achievement suggests that the MyGoals implementation strategies produce the desired improvements in the OTs’ determinants such as knowledge and self-efficacy. These findings are promising because implementers who achieve change objectives are more likely to achieve the ultimate implementation outcomes (11). The implementation strategies were also effective in supporting OTs' delivery of MyGoals. Almost all intervention components were delivered using high-quality empowerment-based approaches. Having good intervention fidelity is one of the ultimate implementation effectiveness outcomes (16).

Informed by the qualitative feedback on the implementation strategies and the MyGoals intervention itself, we identified concrete methods to facilitate the integration of the intervention into real-world settings. We can immediately incorporate some suggestions, especially those related to the delivery methods (e.g., recorded sessions) and *Clinician Education* contents (e.g., promote adaptability) in the next study. However, some suggestions require long-term, complex multi-level implementation efforts. For instance, providing continuing education credit requires an application and approval process as well as financial and human resources. In addition, a systematic approach using adaptation frameworks such as IM Adapt would be necessary to better modify the strategies while maintaining their key ingredients (29, 30). After the modification, it is essential to accurately report the modifications using a systematic guide such as The FRAME-IS (31).

Analysis of the intervention fidelity data revealed further enhancements we can make to the implementation strategies. Additional emphasis and training on the two most omitted components, *Educate and discuss one's current health condition from the biopsychosocial perspective* and *Educate and discuss the benefits of using life goals, goals, and building block goals*, is needed to improve adherence. Participants mentioned that they either forgot or were not sure how to appropriately deliver these components. Notably, *Educate and discuss one's current health condition from the biopsychosocial perspective* was the only component that did not have a script since it needs to be personalized for each client. In the future, we will provide more education and role-playing focused on using this component during *Clinician Education* and add explicit instruction for its implementation to the MyGoals clinician manual. There is evidence suggesting that training can improve general clinicians' communication skills, such as asking open-ended questions and interacting with clients (32, 33). More education and feedback on the use of open-ended questions and explicitly exploring clients' perspectives can be effective to improve competence. We observed that OTs sometimes asked one open-ended question and then did not fully explore clients' perspectives. Clients do not always provide sufficient depth or detail in their initial responses, so it is often necessary to use additional methods or follow-up questions to fully explore their perspectives. In general, we found that individual OTs demonstrated considerably different levels of knowledge, skills, and performance in implementing the MyGoals intervention. Thus, it would be necessary to first understand OTs' existing knowledge, skills, and performance through conversation or self-reflection/evaluation survey before *Clinician Education*. Based on the results of the conversation and survey, the educators of *Clinician Education* can provide additional tailored training modules or Audit & Feedback for personalized support. After building more generalizable data on common weakness areas or concerns of OTs, it would be helpful to develop readily available training modules or educational materials.

This study informs us on how to improve our outcome measures to better capture the mechanisms and outcomes of the MyGoals implementation strategies. We have now added *active listening* and *using guided discovery* to the fidelity assessment. These are key skills in delivering MyGoals, as highlighted in this participant quote: “Having strong active listening skills. That really helps deliver it. Guided discovery and being able to pause to ask an open-ended question and just wait and not jump in and provide options or an answer right away. I think those are really important pieces of delivering it”. We had assumed that these skills were inherent in OTs, so we did not include them in the assessment; however, we observed that this was not the case. Thus, it is necessary to incorporate these items into the fidelity assessment to more thoroughly evaluate competence in MyGoals delivery. We also identified a few change objective survey items that can be improved. For instance, the item, *I acknowledge that current goal setting and goal management practice is not optimal*, was intended to measure if OTs realize that current practice can be improved. However, participants rated this item lower because they did not believe all goal setting and goal management practice is suboptimal. Collaborating with OTs to draft and pilot-test these items will allow us to better evaluate the MyGoals implementation strategies moving forward.

The immediate next step is to incorporate the suggestions of OTs regarding *Clinician Education* and *Audit & Feedback* delivery methods and contents, and study measures, while taking care not to lose the essential elements of MyGoals implementation strategies. Collaboration with OTs is crucial in this process. Moreover, future studies should systematically explore the possible associations between different implementation determinants, such as client-, OT-, organizational-, and policy-level factors, and implementation outcomes. Lastly, to produce more generalizable and replicable evidence on the efficacy of MyGoals implementation strategies and prepare its scale up, advanced study designs such as using both subjective and objective outcome measures, triangulation of different data, blind raters, and diverse participants should be incorporated.

### Limitations

This was a feasibility study with a small number of participants who live in the same geographical area. Multi-level implementation factors may have influenced the current study's findings. For example, organizational and policy-level factors, such as healthcare policy and organizational readiness for implementation, may differ among organizations and affect the study outcomes. The OT-level (e.g., existing knowledge and skills, preferences, and past work experiences) and client-level (e.g., cognitive function, education, goal-oriented personality) determinants may also impact the current implementation outcomes. Logistical limitations prevented the use of blind raters to measure the study outcomes, such as MyGoals intervention fidelity. Some study measures relied on self-report surveys, which may or may not accurately reflect the OTs' actual achievement of the MyGoals implementation strategy change objectives. Further, one participant did not complete all of the study measures. Such limitations can introduce possible bias in the study findings.

## Conclusion

MyGoals implementation strategies are considered to be acceptable, appropriate, and feasible by OTs working in community-based rehabilitation. These strategies may help OTs provide theory-based, client-engaged goal setting and goal management for adults with chronic conditions, and ultimately may improve health in this population. In future research, it will be necessary to advance MyGoals implementation strategies, particularly in terms of streamlined content and various delivery modes, as well as study designs. This can be achieved by including more diverse participants and using advanced study designs to rigorously evaluate the efficacy and effectiveness of the strategies, resulting in more generalizable and replicable evidence.

## Data Availability

The original contributions presented in the study are included in the article/**Supplementary Material**, further inquiries can be directed to the corresponding author.
