# Supplementary material for: Implementation strategies for occupational therapists to advance goal setting and goal management
Source: Front Health Serv. 2023 Jun 7;3:1042029. doi: 10.3389/frhs.2023.1042029 (PMC10282647; doi:10.3389/frhs.2023.1042029)
Supplement: Supplementary file 1 [file Datasheet1.docx]

**Supplementary Material 1. MyGoals manual and client worksheet examples**

**Activity 4. Make My Goal**

**Objective:**

- Guide life goal, goal, building block goal formulation
- Guide the evaluation of self-efficacy and positive outcome expectancy levels of goals
- Educate and discuss the benefits of using different goal types
- Educate clients’ health conditions from the biopsychosocial perspective

Based on the previous activity, guide clients to develop life goals, goals, and building block goals. See definitions, examples, and benefits of three goal types in Fig.4.1. Use the prompts/phrases/questions provided in the following pages to educate clients about different goal types.


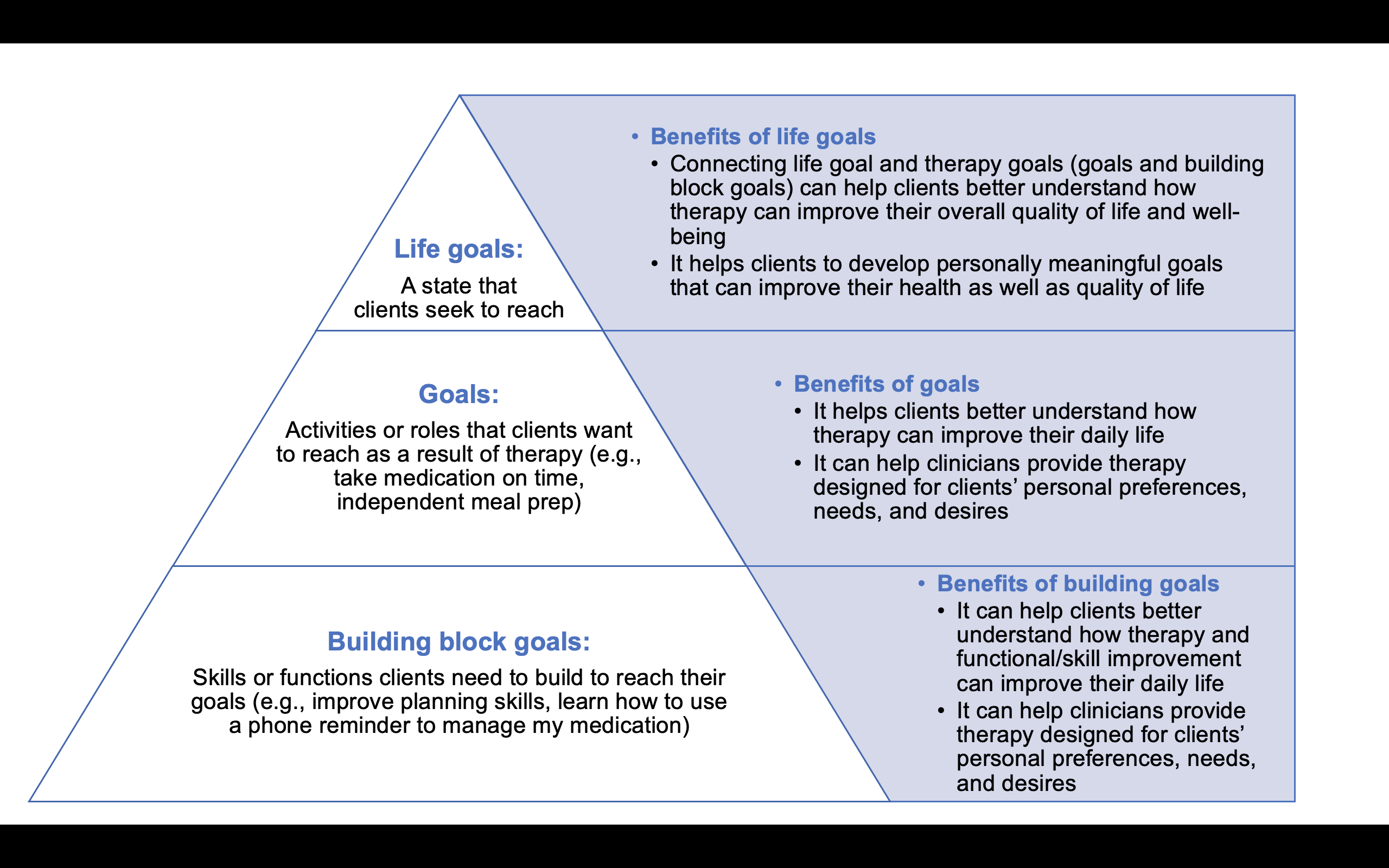


Fig. 4.1 MyGoals pyramid

**MyGoals Goal Pyramid**


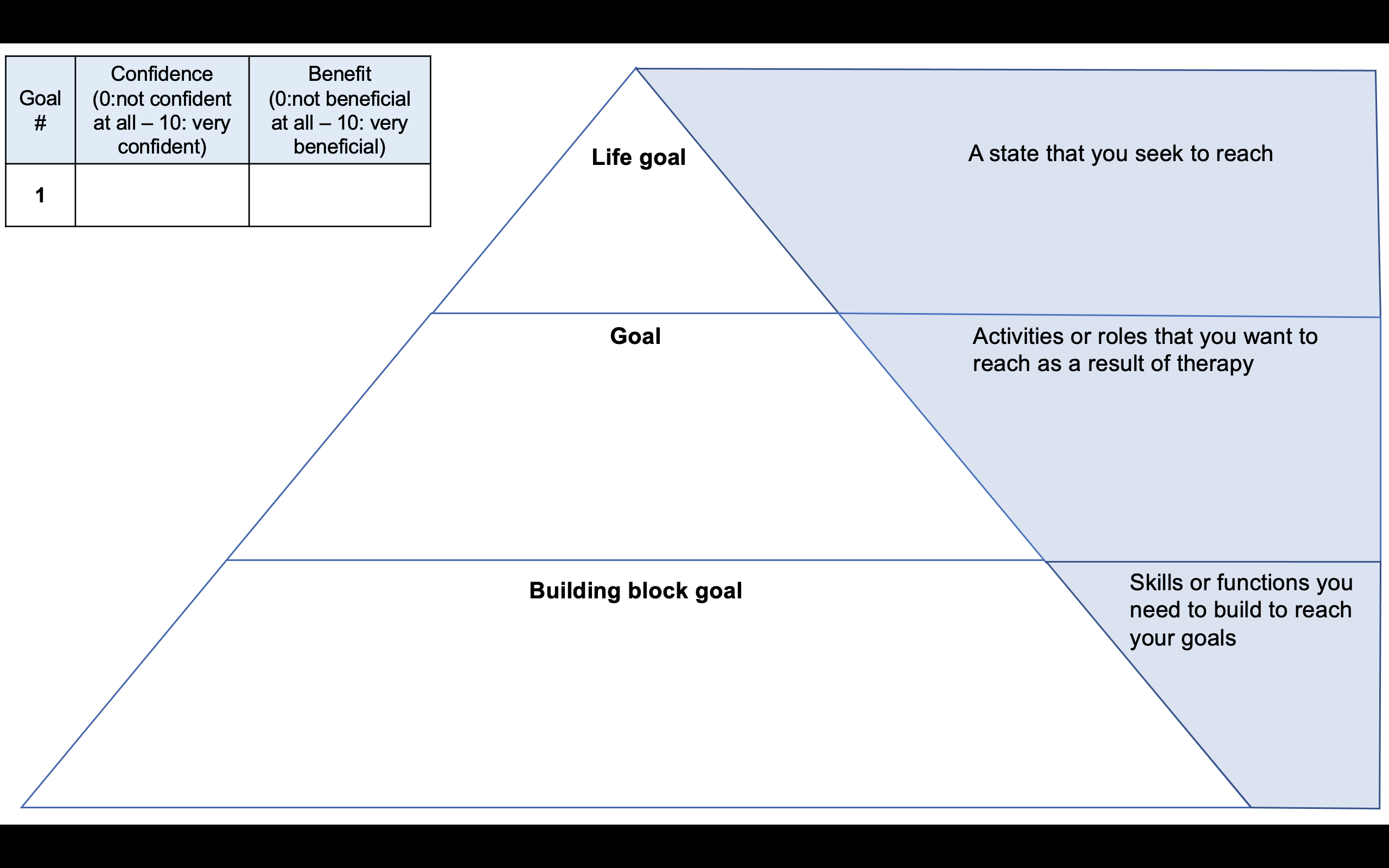


**Activity 4. Make My Goal (cont.)**

1. Provide Find My Goal Sheet to the client.
2. Based on the chosen potential activities or roles, let’s make one goal. Which one do you want to work on?
3. Provide MyGoals Pyramid Sheet to the client.
4. Let’s talk more about your goal to make it more specific. Tell me more about this goal. What do you want to achieve?
5. Write down your goal in the “goal” section of the Pyramid. Make your goal more meaningful and relevant to you using your own words.
6. Given your goal, let’s think about your life goal. A life goal is a state that you seek to reach. It can be about independence, well-being, health, self-image, career, family, relationships, and so on. Life goals can be simple such as *I want to live independently* or as specific as you want.
7. What is your life goal?
8. Write down your life goal in the “life goal” section of the Pyramid.
9. Your life goal may help you see how therapy goals can help you improve your overall quality of life and reach your life goal.
10. Based on your goal, let’s think about your building block goal. Building block goals are skills or functions you need to build to reach your goal. It can be about skills such as planning skills and functions such as walking. What skills or functions do you think can help you to reach your goals? [If clients cannot develop building block goals, provide guidance or examples. These can be explored by using questions about barriers to achieving their goals (e.g., Why is it difficult for you to take medications independently?)]
11. Write down your building block goal in the “building block goal” section of the Pyramid.
12. [Rate confidence to reach each goal] Rate how confident you are in reaching your goal (0: not confident at all – 10: very confident). [If clients have a lower than 7 confidence level, explore what makes them feel less confident. (e.g., Tell me why you rated the confidence 5.) After discussion, if clients are still not confident to reach their goals, consider modifying goals. Do not discourage or force clients to change their goals in this activity. If needed, goals can be revised in Activity 6. It is more important to help clients feel ownership, rather than setting attainable goals.]
13. [Rate perceived potential positive mental, physical, and social outcomes of each goal] Rate how much you may benefit from reaching your goal. Think about the potential positive mental, physical, or social benefits of your goal (0: not beneficial at all – 10: very beneficial). [If clients have a lower than 7 benefit level, explore what makes them see such low benefit (e.g., Tell me about what makes you rate the benefit 5.) Guide clients to see more potential befits of their goals.]
14. [Summarize the goal pyramid] This is your building block goal. [Point to building block goal]. It can help you reach your goal. [Point to the goal] Working towards these goals can help you do your desired activities and reach your life goal [Point to the life goal].
