# Supplementary material for: Implementation strategies for occupational therapists to advance goal setting and goal management
Source: Front Health Serv. 2023 Jun 7;3:1042029. doi: 10.3389/frhs.2023.1042029 (PMC10282647; doi:10.3389/frhs.2023.1042029)
Supplement: Supplementary file 3 [file Datasheet3.docx]

**Supplementary Material 3. Clinician interview guide**

**[Acceptability]**

- What aspects of MyGoals training seem less acceptable?
- How can we make MyGoals training more acceptable?

**[Appropriateness]**

- What aspects of MyGoals training seem less appropriate?
- How can we make MyGoals training more appropriate?

**[Feasibility]**

- What aspects of MyGoals training seem less feasible?
- How can we make MyGoals training more feasible?

**[Intervention change objectives]**

- How can we improve MyGoals training so that it can help you to [understand goal setting and goal management practice concepts and their importance?
